# Legume Plants Enhance the Resistance of Soil to Ecosystem Disturbance

**DOI:** 10.3389/fpls.2017.01295

**Published:** 2017-07-21

**Authors:** Dandan Gao, Xiaoling Wang, Shenglei Fu, Jie Zhao

**Affiliations:** ^1^Key Laboratory of Agro-ecological Processes in Subtropical Region, Institute of Subtropical Agriculture, Chinese Academy of Sciences Changsha, China; ^2^University of Chinese Academy of Sciences Beijing, China; ^3^Key Laboratory of Vegetation Restoration and Management of Degraded Ecosystems, South China Botanical Garden, Chinese Academy of Sciences Guangzhou, China; ^4^College of Environment and Planning, Henan University Kaifeng, China

**Keywords:** legume, understory removal, soil physico-chemical properties, soil biota, resistance index

## Abstract

Cultivation of legume plants is well known to improve soil N level and net primary productivity; besides, it may deliver other ecosystem benefits such as increasing soil carbon sequestration and soil food web complexity. However, little is known about whether legumes can improve the resistance of soils to ecosystem disturbances. In the present study, we compared the resistance of soils to an ecosystem disturbance (understory removal) in the presence or absence of a legume species (*Cassia alata*) in mixed tree species plantations in southern China. Soil physico-chemical and biotic properties were employed to quantify the resistance of soils to understory removal. Our results showed that the resistance indices of soil water content, omnivorous-predacious nematode abundance and nematode channel index to understory removal were greater in the presence of legumes than those without legumes in wet season. The resistance indices of fungal to bacterial ratio, fungivorous nematode abundance and total arthropod abundance were greater in the presence of legume than those without legume species in dry season. Our results indicate that legumes may enhance the resistances of soil physico-chemical and biological properties to the ecosystem disturbance. Our findings could provide a better understanding of the myriad ways in which legumes can positively affect ecosystem functioning.

## Introduction

Forests, including planted forests, provide important ecological services (FAO, 2015), such as soil and water conservation (Keesstra, 2007), soil organic carbon sequestration (Bruun et al., 2015), biodiversity maintenance (Steneck et al., 2002), and functioning biogeochemical cycles (Lucas-Borja et al., 2016). The area of planted forests on Earth as of 2015 is estimated to be 291 million hectares, which accounts for 7.3% of the global forest area (FAO, 2015). This area has been steadily increased since 1990 at the global level and is expected to increase to 300 million hectares by 2020 (FAO, 2010). China has the greatest area of planted forests in the world. Many of the planted forests are under anthropogenic managements to provide wood, fiber, fuel, and non-wood forest product (Wolfslehner and Vacik, 2008). Sustainable management of forests has been widely recognized. Sustainable forest management involves maintaining forest biodiversity, productivity, regeneration capacity, and vitality, which potentially fulfill relevant ecological, economic, and social demands (see Ministerial Council for the Protection of Forests in Europe, 1993). There are plenty of sustainable forest management practices, operations, or strategies that are potentially available to policy makers and farmers. However, no single practice is suitable for all forests (Rice et al., 1997). In addition, inefficient resources exploitation results in severe forest degradation (KissinGer and Herold, 2012; Noriko et al., 2012). Therefore, the suitability of management practices for particular forest ecosystems needs to be evaluated.

Soil fertility degradation is one of the most serious problems that are induced by forest rotation, specifically, relating to nutrient removal through harvests (Van Miegroet et al., 1994; Heilman and Norby, 1998). To a certain extent, fertilization meets the demands of trees for nutrients and maintains the wood production in rotation forests (Binkley and Reid, 1984; Haywood and Tiarks, 1990); but fertilization (especially applied in long term) may induce environmental issues and cause damage to ecosystems, such as groundwater pollution, greenhouse gas emission, soil physico-chemical property change, and soil food webs implification (Barak et al., 1997; Ingerslev, 1997; Heilman and Norby, 1998; Treseder, 2008; Zhao et al., 2014c, 2015a). Concerning these issues, introducing legumes into forest plots is considered as a sustainable forest management practice. Due to the nitrogen (N) fixing ability, many legumes are cultivated to improve soil fertility and are commonly used to increase net primary productivity (NPP) including grain, timber, and forage yield in anthropogenically managed ecosystems (Peoples et al., 1995; Drinkwater et al., 1998; Spehn et al., 2002; Wang et al., 2010). Legume presence has been also reported to increase the soil carbon sequestration (Drinkwater et al., 1998; Fornara and Tilman, 2008; De Deyn et al., 2011; Wu et al., 2016). In addition, the nitrogen-rich litter of the legumes is more readily decomposed by soil microorganisms and this effect can reach high trophic levels through bottom-up control (Gastine et al., 2003; Viketoft et al., 2005, 2009; Zhao et al., 2015b). Specifically, legume could increase the complexity of the soil food webs (Zhao et al., 2014b). However, whether the increased complexity of soil food webs could result in promoting resistance of soil to ecosystem disturbances is poorly known.

Traditionally, understory vegetation is considered to compete for nutrients, water, and light with canopy species (and their seedlings) in forest ecosystems (Raich, 1985; Nambiar and Sands, 1993; Matsushima and Chang, 2007). Therefore, understory vegetation is commonly removed in many forest regions of the world. However, recent studies have revealed that understory vegetation is an important component in many forest ecosystems worldwide and plays significant roles in driving aboveground and belowground ecosystem processes and functions (Nilsson and Wardle, 2005; Zhao et al., 2012; Wan et al., 2014), such as maintaining NPP, aboveground and belowground biodiversity, microclimates and ecosystem nutrient cycling. Understory removal has been reported to be a severe ecosystem disturbance (Zhao et al., 2012) and may not be a sustainable forest management practices (Nilsson and Wardle, 2005; Wu et al., 2011; Wan et al., 2014). However, the understory vegetation is still mowed by the local farmers and forest managers (once- or twice-yearly corresponding to fertilizer application operation and/or logging operation), especially in intensively managed plantations to prevent fire and facilitate forestry operations in southern China and other forest regions of the world (Nilsson and Wardle, 2005; Camprodon and Brotons, 2006; Zhao et al., 2013). In the present study, we explored whether the presence of a legume species could affect the resistance of soil to ecosystem disturbance (i.e., understory removal) in plantations of mixed native tree species in southern China. We hypothesized that legume plants could increase the resistance of soils to understory removal in the forest ecosystems.

## Materials and Methods

### Site Description and Experimental Design

This study was carried out at the Heshan Hilly Land Interdisciplinary Experimental Station (112°50′E, 22°34′N), Chinese Academy of Sciences (CAS), Guangdong Province, China, at an altitude of *c.* 80 m a.s.l. The climate is subtropical monsoon with a distinct wet (from April to September) and dry season (from October to March). The mean annual precipitation and temperature are 1,290 mm and 21.7°C, respectively. The soil is an acrisol (FAO, 2006).

Our experiment was conducted in six mixed plantations of the same age and the similar tree species composition (details see in Zhao et al., 2011, 2013). The most common tree species were *Liquidambar formosana*, *Manglietia glauca*, *Machilus chinensis*, *Cinnamomum burmannii*, and *Michelia macclurel*. The understory vegetation was dominated by *Dicranopteris dichotoma* and sub-dominated by *Miscanthus sinensis*; other common understory species included *Rhodomyrtus tomentosa*, *Melastoma candidum*, *Baeckea frutescens*, and *Clerodendron fortunatum*. Legume species was not recorded in the experimental plots. In March 2007, four plots were established in each of the six plantations (i.e., six replicates). The plots were 10-m × 10-m and each surrounded by a 1-m buffer zone. The treatments include: (i) understory removal; (ii) legume addition; (iii) understory removal + legume addition; and (iv) control (no legume addition and no understory removal). These treatments were assigned randomly to the four plots. The original objective of this experiment was to evaluate the soundness and sustainability of the two forest management practices (i.e., understory removal and legume addition) through monitoring their effects on soil nutrient conditions, soil respiration, and soil biota composition. Empirical evidence showed that understory removal was mainly adverse to soil ecological processes and functions but legume addition tended to be beneficial (Wang et al., 2011; Zhao et al., 2012, 2014b; Wan et al., 2014). Based on these findings, this experiment also provided an opportunity to explore ecosystem resistance to a disturbance (i.e., understory removal). Particularly, the assembly of our four treatments was used to evaluate the effects of understory removal (disturbance) under the absence and presence of legumes, respectively. *Cassia alata*, a shrub-legume species, was selected and added to the understory layer of mixed plantations. The reasons for chosen of *C. alata* are that it can provide large amount of litter with high nitrogen level (Zhang et al., 2009) and is a common and manageable species with high survival rate in the studied area (Ross, 2003; Li et al., 2010; Wang et al., 2011). The saplings of *C. alata* were planted in legume addition plots with a spacing of 1-m × 1-m in March 2007. The shoots of all understory plants in the understory removal plots were removed manually with a machete knife every month. The vegetation cover in control plots was left undisturbed. In addition, there was no other external anthropogenic input to the present ecosystems. Soil sampling was conducted 17 months later to reduce the disturbance effect during implementation processes of legume addition and understory removal.

### Soil Sampling and Analysis

Soil was sampled in August 2008 (wet season) and January 2009 (dry season), respectively. Soil cores were taken at 0–5 cm depth from six randomly selected locations with a steel cylinder (5 cm in diameter) in each plot under each plantation. Six cores from each plot were combined to form one composite sample. The litter above each sampling point was removed gently before the soil core was collected.

Soil pH was determined in 1:2.5 (w/v) soil solutions, and soil water content (SWC %, g of water per 100 g dry soil) was measured by oven-drying for 48 h at 105°C. Soil total nitrogen (g kg^-1^ dry soil) was measured with an ultraviolet spectrophotometer after Kjeldahl digestion, and soil organic carbon (g kg^-1^ dry soil) was determined by dichromate oxidation (Liu, 1996).

Phospholipid fatty acids (PLFA) were analyzed using the method described by Bossio and Scow (1998). Concentrations of each PLFA were calculated based on 19:0 internal standard concentrations. The PLFAs used as fungal biomarkers were 18:2ω6,9; the PLFAs used as bacterial biomarkers were i15:0, a15:0, 15:0, i16:0, 16:1ω7, i17:0, a17:0, 17:0, cy17:0, and cy19:0 (Frostegård and Bååth, 1996; Zelles, 1999; Ruess and Chamberlain, 2010; Zhao et al., 2012). The data of fungal and bacterial PLFA biomarkers were used to calculate the ratio of fungal to bacterial biomass.

Nematodes were extracted from 50 g of fresh soil using the Baermann funnel method (Barker et al., 1985). After fixation in 4% formalin solution, nematodes were counted with an inverted microscope (Eclipse Ts100, Nikon), and the first 100 individuals encountered were identified to genus using a differential interference contrast microscope (ECLIPSE 80i, Nikon). Nematodes were assigned to main trophic groups (bacterivores, fungivores, herbivores, and omnivore-predators) (Yeates et al., 1993) and colonizer-persister scales (Bongers and Bongers, 1998). The nematode data were used to calculate the Shannon–Wiener diversity index, maturity index (MI), plant-parasitic index (PPI), structure index (SI), enrichment index (EI), channel index (CI), and bacterivore index (BaI) (Bongers and Bongers, 1998; Ferris et al., 2001; Neher, 2001; Ferris and Matute, 2003; Zhao et al., 2014a).

The microarthropods were extracted from three additional soil cores (5 cm diameter and 5 cm depth) with a Tullgren funnel (Crossley and Blair, 1991). Fresh soil was placed in the Tullgren funnel, which had a 0.425-mm mesh and which was irradiated with fluorescent lamps (5 W, 15 cm above the soil) for 72 h. Microarthropods that fell through the mesh were collected and stored in vials containing 75% ethanol. Microarthropods were counted with an inverted compound microscope and all the mites and collembolans of each sample were identified to genus level when possible. The microarthropod data were used to calculate the Shannon–Wiener diversity index (Zhao et al., 2014a).

### Data Analysis

A resistance index (RS) was employed to quantify the resistance of soil to understory removal (Orwin and Wardle, 2004) in the wet and dry seasons under two scenarios, i.e., no legume presence (NL) regarding to control and understory removal treatments and legume presence (L) regarding to legume addition and understory removal + legume addition treatments. The RS was calculated as follow:

(1) $$RS(t_{0})=1-\frac{2\times |D_{0}-C_{0}|}{C_{0}+|D_{0}-C_{0}|}$$

where *C*_0_ and *D*_0_ are the values of the non-understory removal disturbed soils (in the control or the legume addition plots) and the understory removal disturbed soils (in the understory removal or the understory removal + legume addition plots) at the end of the disturbance, respectively; and |*D*_0_ – *C*_0_| is the absolute difference between *D*_0_ and *C*_0_. The range of the values of RS is from -1 to +1, a higher RS value indicates greater resistance to disturbance.

Paired-samples *t*-tests were used to compare the differences of the resistance indices between no legume presence scenario and legume presence scenario in the wet and dry seasons. Data were natural log, square root, or rank transformed when required to improve normality and homogeneity of variance. Given the limited replication of the experiment, small sample sizes and short period of experimental time, we highlight *p* < 0.05 as statistically significant and 0.05 < *p* < 0.10 as marginally significant. The paired-samples *t*-test was performed using SPSS software (SPSS Inc., Chicago, IL, United States).

## Results

**FIGURE 1:**
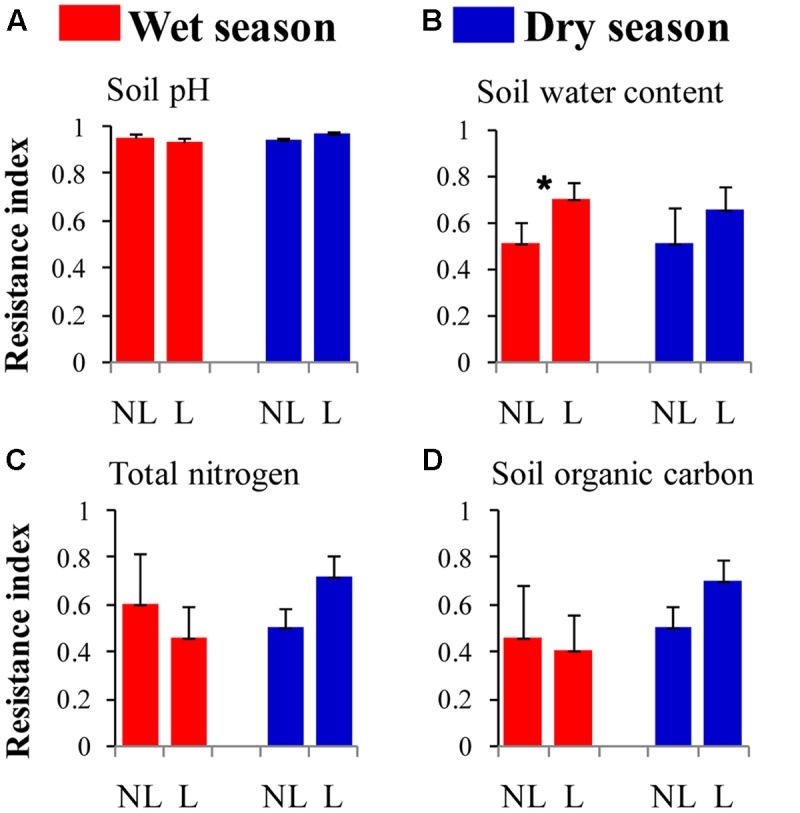
Resistance of soil physico-chemical properties to understory removal in plots with legumes (L) and without legumes (NL) in the wet season and dry season in plantations of mixed native tree species in southern China. **(A)** Soil pH; **(B)** soil water content; **(C)** soil total nitrogen; and **(D)** soil organic carbon. Bars indicate standard errors of means. Within each season, star denotes marginally significant difference between L and NL plots (0.05 < *p* < 0.10).

**FIGURE 2:**
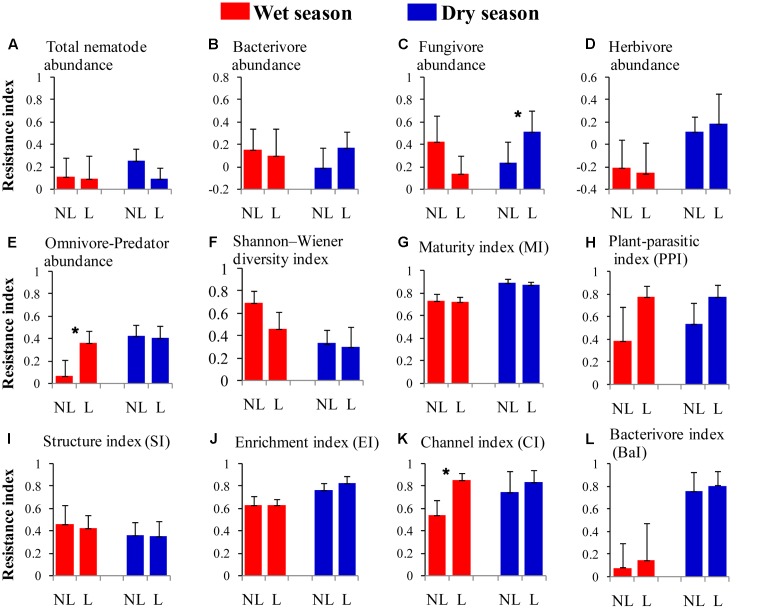
Resistance of soil nematode properties to understory removal in plots with legumes (L) and without legumes (NL) in the wet season and dry season in plantations of mixed native tree species in southern China. **(A)** Total nematode abundance; **(B)** bacterivore abundance; **(C)** fungivore abundance; **(D)** herbivore abundance; **(E)** omnivore-predator abundance; **(F)** Shannon–Wiener diversity index; **(G)** maturity index; **(H)** plant-parasite index; **(I)** structure index; **(J)** enrichment index; **(K)** channel index; and **(L)** bacterivore index. Bars indicate standard errors of means. Within each season, star denotes marginally significant difference between L and NL plots (0.05 < *p* < 0.10).

**FIGURE 3:**
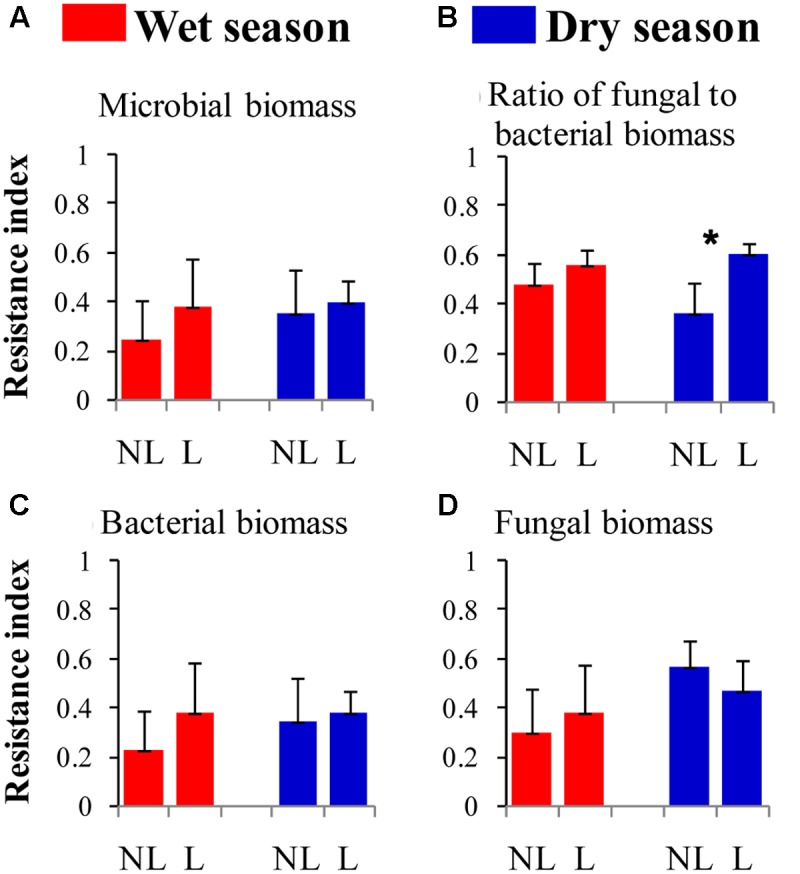
Resistance of soil microbial properties to understory removal in plots with legumes (L) and without legumes (NL) in the wet season and dry season in plantations of mixed native tree species in southern China. **(A)** Soil microbial biomass; **(B)** Ratio of fungal to bacterial biomass; **(C)** Bacterial biomass; and **(D)** Fungal biomass. Bars indicate standard errors of means. Within each season, star denotes marginally significant difference between L and NL plots (0.05 < *p* < 0.10).

**FIGURE 4:**
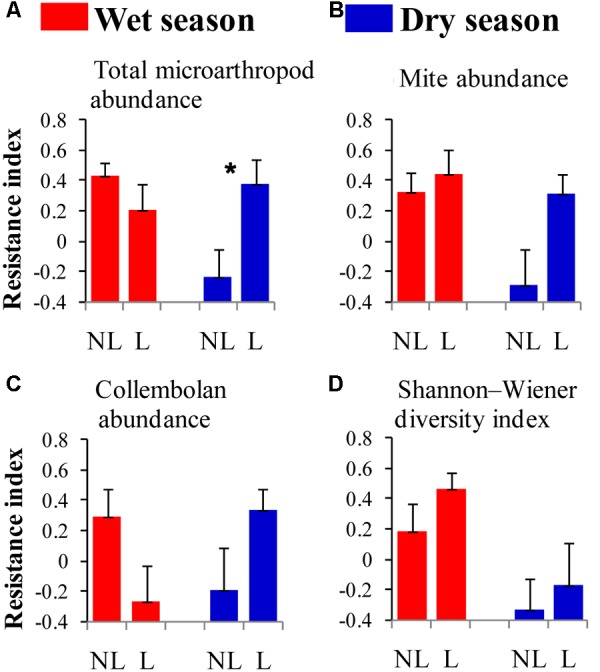
Resistance of soil microarthropod properties to understory removal in plots with legumes (L) and without legumes (NL) in the wet season and dry season in plantations of mixed native tree species in southern China. **(A)** Total microarthropod abundance; **(B)** mite abundance; **(C)** collembolan abundance; and **(D)** Shannon–Wiener diversity index. Bars indicate standard errors of means. Within each season, star denotes marginally significant difference between L and NL plots (0.05 < *p* < 0.10).

The resistance indices for the SWC (*p* = 0.060) (**Figure 1B**), the abundance of omnivorous-predacious nematodes (*p* = 0.080) (**Figure 2E**), and the nematode channel index (*p* = 0.062) (**Figure 2K**) were nearly significantly higher under legume presence than under no legume presence in the wet season; the resistance indices for these variables were not affected by legume presence or not in the dry season (*p* > 0.10). The resistance indices for the ratio of fungal to bacterial biomass (*p* = 0.087) (**Figure 3B**), the abundance of fungivorous nematodes (*p* = 0.058) (**Figure 2C**), and the abundance of total arthropods (*p* = 0.057) (**Figure 4A**) were nearly significantly higher under legume presence than under no legume presence in the dry season; the resistance indices for these variables were not affected by legume presence or not in the wet season (*p* > 0.10). The resistance indices for the other variables of the soil physico-chemical properties, soil microbial communities, soil nematode communities, and soil arthropod communities were not affected by legume presence or not (*p >* 0.10) (**Figures 1**–**4**).

## Discussion

Legume presence significantly increased soil fertility, abundance and/or diversity of soil biota in both seasons except the fungivorous nematode abundance in the wet season (Supplementary Table S1). Understory removal was generally detrimental to soil biota (Supplementary Table S1). All these results confirm the findings of previous studies that understory removal is a severe ecosystem disturbance (Nilsson and Wardle, 2005; Wang et al., 2011; Zhao et al., 2012; Wan et al., 2014) and legume presence is a good forest management practices (Peoples et al., 1995; Drinkwater et al., 1998; Wu et al., 2016). However, ecosytem resistance to disturbance, also known as ecosystem stability, may not depend on high soil nutrient levels, biodiversity, and NPP. For instance, ecosystems with high biodiversity and NPP may not always have high ecological resistance to drought and plant invasion (Palmer and Maurer, 1997; Levine and D’Antonio, 1999; McCann, 2000; Pfisterer and Schmid, 2002). Therefore, a resistance index is highly desirable to be able to quantify stability and provide direct evidence for ecosystem resistance to disturbance (Orwin and Wardle, 2004).

Legume presence tended to increase the resistances of several soil properties to the understory removal in the mixed tree species plantations. The primary reason for the increased resistance of soils to understory removal may be the N-fixing ability of the legumes, which not only increases the soil nitrogen levels but also increases the soil food web complexities (Zhao et al., 2014b). This finding may suggest that legume presence is conducive to resisting ecosystem disturbances, which has important implications for management of forest ecosystems. In other words, adding N-fixing legume species to ecosystems that lacking of legumes is an option to increase ecosystem resistances during managing or conserving natural, semi-natural and non-natural ecosystems. Although a large amount of studies documented the beneficial effects of legumes on soil physico-chemical properties (especially the soil organic carbon and nitrogen contents)(e.g., Peoples et al., 1995; Drinkwater et al., 1998; Fornara and Tilman, 2008; De Deyn et al., 2011; Wu et al., 2016) and several studies reported that legumes increased the abundance and/or diversity of soil biota (e.g., microbes, bacterivorous nematodes, omnivorous-predacious nematodes, epigeic earthworms) (Gastine et al., 2003; Viketoft et al., 2005, 2009; Zhao et al., 2014b, 2015b), the relationships between the legumes and the resistances of the soil properties have not been documented. Only one study reported that legume presence tended to increase ecosystem resistance to plant invasion in a grassland ecosystem at the England site of the BIODEPTH project (Hector et al., 2001), which was consistent with our finding. However, another study reported that legume presences decreased the resistance of aboveground plant biomass production to the drought perturbation in constructed grassland ecosystems at the Swiss site of the BIODEPTH project (Pfisterer and Schmid, 2002), which was contrary to our finding. The likely reason was that the legume species (i.e., *Lotus corniculatus* and *Trifolium pratense*) were more sensitive to drought than most of the plant species at the Swiss site. Compared to the two BIODEPTH studies, the current study tested the resistances of soil properties rather than plant properties to ecosystem disturbances in the presence or absence of legumes and demonstrated that legume presence increased the resistances of more than one soil variable to ecosystems disturbances.

Legume presence tended to increase the resistance of soils to understory removal, which indicated that plots containing legumes suffered less changes (i.e., |*D*_0_ – *C*_0_|) in soil variables under disturbed conditions than did plots without legumes. In other words, legume presence might have reduced the sensitivities of the soils to disturbances. As noted in Zhao et al. (2014b), legume presence enhanced the complexity of soil food webs with more species and more trophic links. This was consistent with the findings of Gastine et al. (2003) and Viketoft et al. (2009, 2005), theyalso demonstrated that legumes enhanced the complexities of the soil food webs. The present study provides direct evidence that legume presence could enhance ecosystem stability. Additionally, this study supports the viewpoint that complex food webs provide functional redundancy and, consequently, may enhance the resistance of soils to disturbance (Ferris et al., 2001; Neutel et al., 2007).

In the present study, our results clearly suggest that the presence of legume plants enhances the resistance of soil to ecosystem disturbances. This finding could provide a better understanding of interactions between legume plants and ecosystem functioning. Particularly, legumes could enhance ecosystem resistance in addition to improve soil fertility. At the global level, the area of planted forest accounts for a substantial portion of total forest area. In planted forestry systems, repeated loss of nutrients from a site is common in the process of forest management (e.g., site preparation and wood harvest). Moreover, planted forestry systems are facing other ecosystem degradation problems such as biodiversity loss, pest and disease damage, fire, air pollution, and extreme climate. Therefore, our finding has at least one implication that adding nitrogen fixing legume plants to the forest ecosystems is a potential good management practice. From another point of view, investigation of the effects of legume removal on ecosystem processes and functions in future studies may be conducive to the knowledge of the myriad ecological functions of legumes species. Additionally, the effects of intensities of understory removal (e.g., application frequency) and legume addition or removal (e.g., legume density) need to be determined in future studies.

## Author Contributions

SF designed the experiments; JZ, DG, and XW performed the experiments; DG and JZ carried out data analysis and wrote the manuscript. SF helped in preparing the manuscript and in interpretation of the analyses during constructive discussions.

## Conflict of Interest Statement

The authors declare that the research was conducted in the absence of any commercial or financial relationships that could be construed as a potential conflict of interest.
