# Supplementary material for: Legume Plants Enhance the Resistance of Soil to Ecosystem Disturbance
Source: Front Plant Sci. 2017 Jul 21;8:1295. doi: 10.3389/fpls.2017.01295 (PMC5519628; doi:10.3389/fpls.2017.01295)
Supplement: Supplementary file 1 [file Table_1.DOCX]

Table S1. The effect of legume presence (LP), understory removal (UR), and their interactions on soil physico-chemical properties, soil microbial properties, soil nematode properties, and soil microarthropod properties in the wet and dry seasons.

| Soil properties | Wet season | | | | | | | |  | Dry season | | | | | | | |
| --- | --- | --- | --- | --- | --- | --- | --- | --- | --- | --- | --- | --- | --- | --- | --- | --- | --- |
|  | LP | |  | UR | |  | LP×UR | |  | LP | |  | UR | |  | LP×UR | |
|  | *F* | *p* |  | *F* | *p* |  | *F* | *p* |  | *F* | *p* |  | *F* | *p* |  | *F* | *p* |
| pH | 0.133 | 0.719 |  | 0.802 | 0.382 |  | 0.485 | 0.495 |  | 0.088 | 0.770 |  | **4.933** | **0.038-** |  | 0.789 | 0.385 |
| Soil water content | 2.024 | 0.172 |  | 2.036 | 0.171 |  | 0.041 | 0.841 |  | 0.994 | 0.331 |  | 0.730 | 0.403 |  | **3.213** | **0.088** |
| Total nitrogen | **8.308** | **0.010+** |  | 0.234 | 0.635 |  | 0.018 | 0.894 |  | 0.611 | 0.444 |  | 2.102 | 0.163 |  | 0.353 | 0.559 |
| Soil organic carbon | **5.462** | **0.031+** |  | 0.084 | 0.775 |  | 0.251 | 0.623 |  | 0.609 | 0.444 |  | 2.557 | 0.125 |  | 0.005 | 0.944 |
| Microbial biomass | 1.698 | 0.209 |  | 0.005 | 0.945 |  | 0.272 | 0.608 |  | 0.000 | 0.986 |  | 0.424 | 0.522 |  | 0.017 | 0.896 |
| Bacterial biomass | 0.957 | 0.341 |  | 2.216 | 0.154 |  | 0.048 | 0.829 |  | 0.790 | 0.385 |  | 1.969 | 0.176 |  | 0.010 | 0.922 |
| Fungal biomass | 1.723 | 0.206 |  | 0.001 | 0.980 |  | 0.321 | 0.578 |  | 0.002 | 0.962 |  | 0.341 | 0.566 |  | 0.018 | 0.896 |
| Ratio of fungal to bacterial biomass | 0.179 | 0.677 |  | **10.66** | **0.004-** |  | 2.059 | 0.168 |  | 1.041 | 0.320 |  | 1.779 | 0.197 |  | 0.377 | 0.546 |
| Total nematode abundance | 1.377 | 0.256 |  | 0.000 | 0.987 |  | 0.009 | 0.927 |  | 0.001 | 0.972 |  | **9.488** | **0.006-** |  | 0.333 | 0.570 |
| Bacterivore abundance | 0.825 | 0.376 |  | 0.044 | 0.835 |  | 0.082 | 0.777 |  | 2.472 | 0.132 |  | 0.071 | 0.793 |  | 0.001 | 0.982 |
| Fungivore abundance | **8.138** | **0.011-** |  | 0.000 | 0.998 |  | 2.67 | 0.120 |  | 0.457 | 0.507 |  | 0.936 | 0.345 |  | 1.049 | 0.318 |
| Herbivore abundance | **10.65** | **0.004+** |  | 2.146 | 0.160 |  | 2.999 | 0.100 |  | 2.442 | 0.134 |  | 0.381 | 0.544 |  | 2.239 | 0.150 |
| Omnivore-Prdator abundance | 1.678 | 0.212 |  | **3.396** | **0.082-** |  | 0.127 | 0.726 |  | 0.000 | 0.983 |  | **6.218** | **0.022-** |  | 0.274 | 0.607 |
| Nematode Shannon–Wiener index | 1.643 | 0.216 |  | 0.201 | 0.659 |  | 0.814 | 0.379 |  | 0.131 | 0.722 |  | 0.332 | 0.571 |  | 0.229 | 0.637 |
| Maturity index (MI) | 0.539 | 0.472 |  | 0.304 | 0.588 |  | 0.31 | 0.584 |  | 0.007 | 0.934 |  | **4.164** | **0.055-** |  | 0.006 | 0.938 |
| Plant-parasite index (PPI) | 0.268 | 0.611 |  | 1.823 | 0.194 |  | 0.136 | 0.717 |  | **3.546** | **0.074+** |  | 1.745 | 0.201 |  | **5.663** | **0.027** |
| Structure index (SI) | 0.129 | 0.724 |  | 0.374 | 0.548 |  | 0.089 | 0.769 |  | 0.000 | 0.991 |  | **3.165** | **0.09-** |  | 0.073 | 0.790 |
| Enrichment index (EI) | 2.794 | 0.112 |  | **4.095** | **0.058-** |  | 2.498 | 0.131 |  | 0.015 | 0.904 |  | 0.414 | 0.527 |  | 0.284 | 0.600 |
| Bacterivore index (BaI) | 1.874 | 0.188 |  | 1.424 | 0.248 |  | 2.819 | 0.110 |  | 0.276 | 0.605 |  | 0.001 | 0.971 |  | 0.499 | 0.488 |
| Channel index (CI) | **5.698** | **0.028+** |  | 0.062 | 0.806 |  | 0.601 | 0.448 |  | 0.195 | 0.663 |  | 0.438 | 0.516 |  | 0.218 | 0.645 |
| Total microarthropod abundance | 0.548 | 0.469 |  | 1.993 | 0.175 |  | 0.035 | 0.854 |  | 0.621 | 0.440 |  | **3.738** | **0.067-** |  | 0.065 | 0.801 |
| Mite abundance | 0.338 | 0.568 |  | **3.108** | **0.095-** |  | 0.028 | 0.868 |  | 0.497 | 0.489 |  | **3.078** | **0.095-** |  | 0.007 | 0.935 |
| Collembolan abundance | 0.168 | 0.687 |  | 2.211 | 0.154 |  | 0.047 | 0.831 |  | 0.341 | 0.566 |  | 2.013 | 0.171 |  | 0.007 | 0.934 |
| Arthropod Shannon–Wiener index | **3.253** | **0.088+** |  | 0.005 | 0.945 |  | 0.087 | 0.771 |  | 0.719 | 0.407 |  | 1.443 | 0.244 |  | 0.393 | 0.538 |

- and + indicate significant negative and positive effect of LP or UR on the soil properties, respectively (*p*<0.10).
